# Resveratrol protects human lens epithelial cells against H_2_O_2_-induced oxidative stress by increasing catalase, SOD-1, and HO-1 expression

**Published:** 2010-08-04

**Authors:** Yi Zheng, Yaohua Liu, Jinying Ge, Xiaoyuan Wang, Lijuan Liu, Zhigao Bu, Ping Liu

**Affiliations:** 1 Department of Ophthalmology, The First Affiliated Hospital of Harbin Medical University, Harbin, P.R. China; 2 Department of Neurosurgery, The First Affiliated Hospital of Harbin Medical University, Harbin, P.R. China; 3 National Key Laboratory of Veterinary Biotechnology, Harbin Veterinary Research Institute, Chinese Academy of Agricultural Sciences, Harbin, P.R.China

## Abstract

**Purpose:**

Oxidative damage induced by H_2_O_2_ treatment can irreversibly damage the lens epithelium, resulting in cell death and cataract. Whether the effects of oxidative stress could be attenuated in cultured human lens epithelial cells by incubation with resveratrol (RES) is still unknown. In the present study, we examined the function of resveratrol in protecting human lens epithelial B-3 (HLEB-3) cells against H_2_O_2_ induced cell death and cell apoptosis, its role in reducing H_2_O_2_ induced intracellular reactive oxygen species (ROS) accumulation, and investigated the mechanism by which resveratrol underlies the effect.

**Methods:**

HLEB-3 cells, a human lens epithelial cell line, were exposed to 100 μM H_2_O_2_ with or without RES pre-treatment at different concentrations for different time duration. Cell viabilities were monitored by 4-[3-[4-iodophenyl]-2-4(4-nitrophenyl)-2H-5-tetrazolio-1,3-benzene disulfonate] (WST-1) assay. The apoptosis rate and ROS generation were detected by flow cytometric analysis. Expression levels of superoxide dismutases-1 (SOD-1), catalase, and heme oxygenase-1 (HO-1) proteins were measured by western-blotting analysis. p38 and c-jun N terminal kinase (JNK) activation was also evaluated by western-blotting analysis.

**Results:**

Resveratrol clearly reduced H_2_O_2_ induced cell apoptosis and ROS accumulation; protected HLEB-3 cells from H_2_O_2_ induced oxidative damage, and increased the expression levels of SOD-1, catalase, and HO-1. Further studies showed that RES also inhibited H_2_O_2_ induced p38 and JNK phosphorylation.

**Conclusions:**

These findings suggested that RES protected HLEB-3 cells from H_2_O_2_ induced oxidative damage, presumably by inducing three antioxidative enzymes including catalase, SOD-1, and HO-1.

## Introduction

There is significant evidence that oxidative damage acts as a major factor in the initiation and progression of numerous age-related diseases, such as Alzheimer and Parkinson diseases, age related macular degeneration, and age-related cataract [1]. The transparent ocular lens is especially sensitive to oxidative damage because the fiber cells of the lens are not renewed and have to last a lifetime. Damage to these cells results in degradation of protein and ultimately inducing age-related cataract. Therefore the eye lens has evolved a wide variety of protective and repair systems to defend oxidative stress, including high levels of reduced glutathione (GSH) [2] and abundant antioxidant enzymes such as superoxide dismutases-1 (SOD-1) and catalase [3]. Aging of the lens is characterized by diminishing levels of these systems [2,3] whose loss is the leading cause of cataract formation [4,5]. Several epidemiological observations have suggested that acute reactive oxygen species (ROS) induced by H_2_O_2_ treatment can irreversibly damage the lens epithelium, resulting in cell death and cataract [6]. Artificial targeting of catalase to the mitochondria deferred cataract formation in mice [7], which suggested that scavenging of mitochondrial H_2_O_2_ is important for lens maintenance and delaying of cataract formation. Therefore finding strategies for protecting lens epithelial cells from oxidative stress induced cytotoxicity is an important objective.

In recent years, great attention has been paid on natural dietary antioxidants especially polyphenols which are important for counteracting oxidative stress [8,9]. Resveratrol (3, 5, 4'-trihydroxystilbene; RES) is a phytoalexin polyphenolic natural compound found in several plants, including grapes, peanuts, pines and their related products. It has proved to be a critical matter with antioxidant functions in vitro and in cell culture models [10]. Ungvari et al. [11],  found that RES treatment upregulated the expression of glutathione peroxidase, catalase, and heme oxygenase-1 (HO-1) in cultured arteries, and it seems to increase vascular oxidative stress resistance by scavenging H_2_O_2_ and preventing endothelial cell against oxidative stress-induced cell death. The King et al. [12],  study suggested that RES significantly reduced basal and H_2_O_2_ induced intracellular ROS accumulation, therefore reduced the need for RPE cells to elicit an oxidant-induced survival response then decreased H_2_O_2_ induced extracellular signal regulated kinase (ERK_1/2_) activation in retinal pigment epithelial (RPE) cells. Although observations demonstrated the broad antioxidant activity of RES and showed that it was an effective scavenger of ROS [13-16], whether it has some role in preventing against H_2_O_2_ induced oxidative stress in human lens epithelial cells and the mechanisms of this effect have not been documented.

In the present study we first examined whether resveratrol could reduce H_2_O_2_ induced cell apoptosis and cell death in cultured human lens epithelial B-3 (HLEB-3) cells; then we detected if RES can scavenge intracellular ROS accumulation; finally we investigated the mechanism of RES in protecting the HLEB-3 cells from oxidative damage.

## Methods

### Materials

HLEB-3 cells (human lens epithelial-B3 cells) were obtained from the ATCC (Rockville, MD). Fetal bovine serum (FBS) and Dulbecco’s modified Eagle’s medium (DMEM) were obtained from Gibco (Grand Island, NY). Propidium Iodide (PI) and Annexin-V were obtained from Becton Dickinson (Mountain View, CA). Resveratrol was from Sigma Chemical Co. (St. Louis, MO). Anti-JNK, anti-phosphorylation of JNK (p-JNK), anti- phosphorylation of P38 (p-p38) (Thr180/Tyr182), anti-p38, anti-catalase, anti-SOD-1, and anti-HO-1 antibodies were purchased from Santa Cruz Biotechnology Inc. (Santa Cruz, CA).

### Cell culture

HLEB-3 cells were cultured in DMEM supplemented with heat-inactivated (56 °C, 0.5 h) 15% FBS at 37 °C in a humidified atmosphere of 5% CO_2_. The cells were seeded in a 60 mm culture dish (Falcon; Becton Dickinson). When grown to 75%–80% confluence, the cells were treated with the indicated concentration of H_2_O_2_ for the required time or pretreated with RES for different time before the H_2_O_2_ treatment. At the indicated time points, the cells were collected for different assays.

### Cell viability assay

The proliferation of HLEB-3 cells was evaluated by using a sulfonated tetrazolium salt WST-1 (4-[3-[4-iodophenyl]-2–4(4-nitrophenyl)-2H-5-tetrazolio-1,3-benzene disulfonate]). The measurement is based on the ability of viable cells to cleave tetrazolium salts by mitochondrial dehydrogenases. Briefly, HLEB-3 cells were plated at a density of 1×10^4^ cells/well in 96-well microplates, in DMEM containing 15% FBS and incubated at 37 °C in a humidified atmosphere of 5% CO_2_. After a 24-h incubation, at approximately 70% confluence, cells were pretreated with 2.5, 5.0, 10.0, and 20.0 µM RES for 12 h, 24 h, and 48 h. After incubation for the indicated time, cells were treated with 50, 100, and 200 µM H_2_O_2_ in combination for 24 h. To assay for cell viability, 10 µl/well WST-1 reagent was added and incubated for 2 h at 37 °C and 5% CO_2_. For HLEB-3 cells, the absorbance of the samples was measured at 450 nm by using a microplate reader with a background control as the blank. The cell survival ratio was expressed as percentage of the control.

### Flow cytometric analysis using annexin V and propidium iodide (PI)

Cells were grown on a six-well plate at 1×10^5^ cells per plate and pretreated with or without different concentration of RES for 12 h before 100 μM H_2_O_2_ treatment for 24 h. Cells were centrifuged to remove the medium, washed with cold 1× PBS, and stained with annexin V-FITC and PI in binding buffer (10 mM Hepes, 140 mM NaCl, and 2.5 mM CaCl_2_). Ten thousand events were collected for each sample. Stained cells were analyzed using Cell-Quest software in the FL1-H and FL2-H channels.

### ROS detection

The production of reactive oxygen species (ROS) was monitored using flow cytometry. Cells were incubated with 10.0 μM 5-(and-6)-carboxy-20, 70-dichlorodihydrofluorescein diacetate (carboxy-H2DCFDA) C-400 (Molecular Probes, Eugene, OR) for 30 min. They were then washed and incubated with complete medium for 2 h. ROS generation was determined using a FAC Scan flow cytometry using Cell-Quest software (Becton Dickinson) and fluorescent signals were displayed as histograms.

### Western blotting

After washing with ice-cold PBS, cells were lysed by adding 200 μl of RIPA buffer (100 mM NaCl, 2 mM EDTA, 1 mM PMSF, 1% NP-40 and 50 mM Tris-HCl [pH 7.2]). Cell lysates were collected and their protein concentration was evaluated using a protein assay (Bio-Rad, Melville, NY).The lysates (20 μg per lane) were separated by 10%–15% SDS–PAGE gels and then transferred to polyvinylidene difluoride (PVDF) membranes (Millipore, Bedford, MA) at 20 V for 50 min. Membranes were soaked in 5% BSA (Sigma) overnight. The membranes were incubated with primary antibodies overnight at 4 °C, and thereafter incubated with the corresponding peroxidase-linked secondary antibodies (Amersham, Santa Cruz, CA) for 1 h at room temperature. Signals were developed by a standard enhanced chemiluminescence method following the manufacturer’s protocol (Amersham).

### Statistical analysis

Statistical analysis was evaluated using Student’s *t*-test (SPSSs program version 10.1; SPSS, San Rafael, CA). A p<0.05 was considered statistically significant.

## Results

### Effects of resveratrol on protecting against H_2_O_2_-induced cell death and apoptosis in HLEB-3 cells

**Figure 1:**
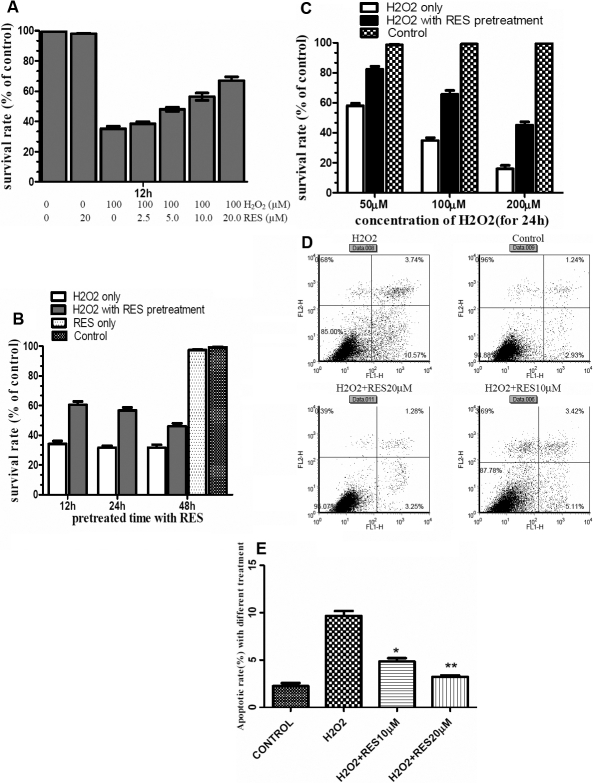
Resveratrol protects against H_2_O_2_-induced cell death and apoptosis in HLEB-3 cells. **A**: The HLEB-3 cells were incubated with different concentrations of RES (2.5, 5.0, 10.0, and 20.0 μM) for12 h before H_2_O_2_ (100 μM) treatment for 24 h. **B**: The cells were incubated with 20.0 μM RES for different time (12, 24, 48 h), then treated with 100 μM H_2_O_2_ for 24 h. **C**: The cells were pretreated with 20.0 μM RES for 12 h,then exposed to different concentration of H_2_O_2_ (50, 100, 200 μM) for 24 h, the cell viability was measured by WST-1 assay. The data are represented as means±SD from three independent experiments. The asterisk indicates that p<0.05 compared to the untreated control. **D**: The cells were incubated with 10.0 and 20.0 μM RES for 12 h then treated with 100 μM H_2_O_2_ for 24 h. Flow cytometric analysis was used to quantify the rate of cell apoptosis using double staining of Annexin V-FITC and PI. The result is one representative example of three separate experiments. **E**: Resveratrol (20μM) significantly decreased the apoptosis rate of HLEB-3 cells, **p<0.01 versus positive control (*t*-test), the preventative effect of 20 μM RES was improved versus 10 μM RES,*p<0.05.

To investigate whether RES confers protection against H_2_O_2_ induced cell damage in HLEB-3 cells, we detected the cell viability of HLEB-3 cells after 100 μM H_2_O_2_ incubation for 24 h, with or without RES pretreatment at multiple concentrations (2.5, 5.0, 10.0, and 20.0μM) for 12h. The results showed that pretreatment with RES prevented 100 μM H_2_O_2_ induced loss of cell viability by a dose dependant manner, as shown in Figure 1A. Thus 20 μM was determined as the optimal dose for RES pretreatment. Then we investigated whether incubation time of RES had any difference on the protective effect against H_2_O_2_ induced cell death. WST-1 assay showed that pretreatment with RES for 12 h was the optimal time compared with others as Figure 1B shows. Then we selected a 12 h pretreatment with 20.0 μM RES before exposure to different concentrations of H_2_O_2_ and found that the protecting effect of RES was proportional to H_2_O_2_ levels as Figure 1C shows. Taken together, these results showed that RES had a protective role against H_2_O_2_ induced cell damage dose-dependantly and the optimal incubation time for RES pretreatment is 12 h.

To examine whether RES protects against H_2_O_2_-induced apoptosis, the HLEB-3 cells were incubated with 10.0 μM and 20.0 μΜ RES for 12 h then treated with 100 μM H_2_O_2_ for 24 h. Flow cytometric analysis was used to quantify the rate of cell apoptosis using double staining of Annexin V-FITC and PI. Significant apoptosis was observed in HLEB-3 cells treated with 100 μM H_2_O_2_ while RES pretreatment at 10.0 μM and 20.0 μM clearly decreased H_2_O_2_ induced apoptosis (Figure 1D). Three repeated experiments showed RES pretreatment dose-dependantly decreased the apoptosis ratio induced by H_2_O_2_, which was statistically significant (Figure 1E).

### Resveratrol reduced the generation of reactive oxygen species (ROS) induced by H_2_O_2_ in HLEB-3 cells

**Figure 2:**
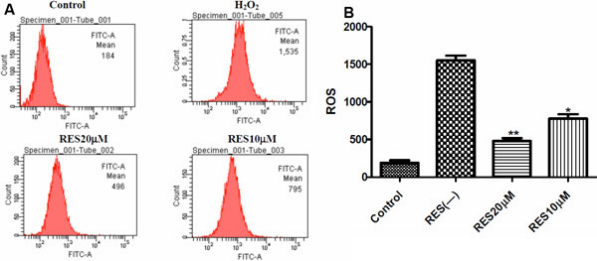
Resveratrol reduced the generation of ROS. **A**: The cells were pretreated with 10.0 and 20.0 μΜ RES for 12 h followed by treatment with 100 μM H_2_O_2_ for 2 h. The production of ROS was examined by measuring the level of ROS production using DCF-DA by flow cytometry. The result is one representative example of three separate experiments. **B**: Resveratrol (20.0 μM) significantly reduced the ROS generation in HLEB-3 cells **p<0.01 versus the RES negative control. The degree of 20.0 μM RES was higher than 10.0 μM RES *p<0.05.

The ROS induced by H_2_O_2_ was examined by measuring the level of ROS production using DCF-DA. HLEB-3 cells treated with 100 μM H_2_O_2_ for 2 h resulted in the production of ROS with an approximately twofold increase compared to nontreated cells. However, the cells pretreatment with RES markedly reduced the ROS levels generated by H_2_O_2_ in HLEB-3 cells (Figure 2A). Three repeated experiments showed RES pretreatment dose-dependently inhibited ROS generation induced by H_2_O_2_, which had statistical significance (Figure 2B).

### Resveratrol upregulated the expression levels of SOD-1, HO-1, and catalase

**Figure 3:**
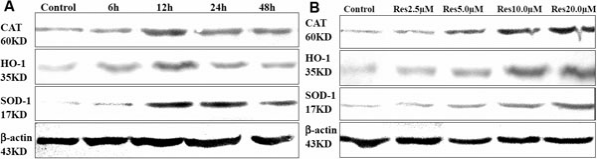
Resveratrol upregulated the expression of SOD-1, HO-1, and catalase. **A**: western blotting analysis of SOD-1, HO-1, and catalase at different time points (6, 12, 24, and 48 h) after pretreatment with 20.0 μM resveratrol. **B**: western blotting analysis of SOD-1, HO-1, and catalase 12 h after pretreatment with different concentration of resveratrol (2.5, 5.0, 10.0, and 20.0 μM). The expression levels of RES pretreated cells significantly increased compared with control cells (p<0.05), and the expression levels were proportional to the concentration of resveratrol.

Since RES can significantly reduce the ROS levels and protect against H_2_O_2_ induced cell death, we presumed that RES may induce some enzymes which can protect the cells against cytotoxicity induced by H_2_O_2_. We evaluated the expression levels of three enzymes by western-blot analysis in treated HLEB-3 cells, which are known or believed to be essential in oxidative stress protection, including superoxide dismutases (SODs), heme oxygenase (HO-1), and catalase. We detected the expression levels of these proteins at 6, 12, 24, and 48 h after the initial treatment with 20.0 μM RES. We found that as time went by, the protein levels of pretreated cells were increased compared with control cells, especially at 12 h, as Figure 3A shows. Consistent with these results, the WST-1 assay showed that pretreatment with RES for 12 h was the optimal time compared with others. Then we examined the protein levels of these enzymes at 12 h after pretreatment with 2.5, 5.0, 10.0, and 20.0 μM RES and found that the expression levels of these proteins were proportional to the concentration of RES, as Figure 3B shows. These results indicated that SOD-1, HO-1, and catalase may play an important role in the protective action of RES.

### Resveratrol inhibited p38 and JNK phosphorylation

**Figure 4:**
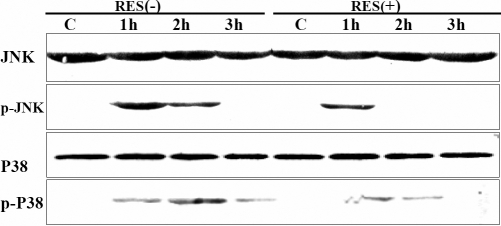
Resveratrol inhibits p38 and JNK phosphorylation. HLEB-3 cells were treated with 100 μM H_2_O_2_ with or without 20.0 μM resveratrol for intervals ranging between 1 and 3 h and p38 and JNK activities were evaluated by western blotting analysis using anti-phosphorylated p38 and anti-phosphorylated JNK antibodies. Resveratrol significantly inhibited p38 and JNK phosphorylation compared to control. Anti-p38 and anti-JNK protein antibodies show equal loading of protein samples.

To gain further proof on the protective effect of RES, we studied the effect of RES on the pathways potentially activated during apoptosis. It is generally accepted that ERK activation is essential for cell survival, whereas activation of JNK and p38 is thought to play an important role in cell death [17,18]. Therefore, we investigated whether the p38 and JNK pathways were inhibited by treatment with RES. HLEB-3 cells were treated with 100 μM H_2_O_2_ with or without RES pretreatment. Whole cell extracts were prepared and analyzed using antibodies against the active phosphorylated forms of JNK and p38. As shown in Figure 4, the peak phosphorylation of JNK (MW 46 kDa) occurred 1 h after H_2_O_2_ treatment and the activation was maintained for up to 1–2 h. After pretreatment with RES, although H_2_O_2_ induced phosphorylation of JNK occurred at 1 h, it quickly disappeared and returned to non-activation form at 2 h. Phosphorylation of p38 was activated at 1 h and peaked at 2 h, and was sustained for up to 3 h after H_2_O_2_ treatment. However, after pretreatment with RES, the peak time of phosphorylation of p38 still occurred at 1 h, but decreased and only maintained for 2 h. Moreover, pretreatment with RES, both p-p38 and p-JNK expression levels were decreased compared with the un-pretreated group. These results suggested that RES pretreatment suppressed JNK and p38 phosphorylation.

## Discussion

Our results show that RES actually protects HLEB-3 cells from H_2_O_2_ induced oxidative damage and the protective mechanism is that RES induces three enzymes thought to be essential in oxidative stress protection (SOD-1, HO-1, and catalase). Although several studies have found that RES can attenuate the processes that associated with cataractogenesis as well as inhibit cataract formation in rodents [18-21], our experiment is the first to show its protective function in human lens epithelial cells and its mechanism against oxidative stress. Thus it has a potential therapeutic role in the prevention of cataract.

Wan-Cheng et al. [22] found that cataract patients from 12 to 94 years old had apoptotic epithelial cells ranging from 4.4 to 41.8%. By contrast, in eight normal human lenses of comparable age, very few apoptotic epithelial cells were observed. This result indicated that this single layer of lens epithelial cells is essential for maintaining the metabolic homeostasis and transparency of the entire lens [23]. If such conditions are altered or disturbed by factors such as oxidative stress, the viability of the lens epithelial cells may be jeopardized, possibly resulting in opacification of the lens. Furthermore, previous studies demonstrated that chronic exposure to light, and the pathogenic activities of lens epithelial cells can produce high local oxygen concentrations (ROS), that is rich in the lens [24]. The oxidative stress induced by ROS has been recognized as an important mediator of apoptosis in lens epithelial cells [6,25,26], which had been identified as providing an important molecular basis for both the initiation and the progression of cataract [27,28]. Therefore, in our experiment H_2_O_2_ was used as the oxidant model of classical oxidative stress. H_2_O_2_ treated cells showed an increased production of intracellular ROS and a high apoptosis rate. However RES pretreatment can significantly inhibit the ROS production in H_2_O_2_ treated HLEB-3 cells, which suggested a primary role of RES in protection against ROS. Moreover, flow cytometry analysis using PI and annexin V showed that H_2_O_2_ induced cell apoptosis in HLEB-3 cells were significantly reduced by RES pretreatment. These results indicated that RES can not only reduce the production of ROS, but also prevent HLEB-3 cells from apoptosis caused by H_2_O_2_, thus conferring a protective effect against H_2_O_2_ induced cell damage.

Previous studies showed that disruption of the balance between ROS production and scavenging often lead to apoptotic cell death, which is associated with cataract formation [6,29-33]. Therefore, cellular defenses have been proposed to be important in protecting the lens epithelial cells against oxidative stress and reducing the progression of various types of cataract [34]. Based on the results of this study, we speculated that RES may induce some enzymes in HLEB-3 cells which scavenge the reactive oxygen radical productions induced by H_2_O_2_. Among these antioxidant enzymes, catalase appears to be crucial, as it can eliminate H_2_O_2_ directly. Furthermore, we also detected other two enzymes which were essential in oxidative stress protection, SOD-1 and HO-1. Previous studies suggested that heme oxygenase enzymic activity removed the prooxidant molecule heme and generated the free radical scavengers biliverdin and bilirubin, therefore HO-1 was usually regarded as an antioxidant-inducible cellular defense [35]. Therefore, we detected the expression of these three enzymes through western-blot analysis; the results demonstrated that there was an increase in catalase, SOD-1, and HO-1 expression at the protein level. Moreover, induction of those molecules were greatest after 12 h of RES incubation at 20 uM compared with other time durations and concentrations, which was positively correlated with the protective effects of RES. Thus it is indicated that the induction of catalase, SOD1, and HO-1 is involved in the mechanism of RES protective effects.

Several investigations reported that RES reduced the proliferation of various cells via inhibition of the ERK_1/2_ and MAPK signaling cascade [36-41]. It is universally accepted that ERK activation is necessary for cell survival, whereas activation of JNK and p38 is thought to play an important role in cell death signaling [17,42]. In the present study, for the sake of further proof on the protective effect afforded by RES against oxidative stress, we examined the expression of two major signaling proteins in the MAPKs pathways. Our data showed that H_2_O_2_-induced p38 and JNK activation was significantly reduced when cells were pretreated with RES. These results suggested that RES reduced H_2_O_2_ induced intracellular ROS accumulation, decreased the intracellular oxidative level, indirectly prevented HLEB-3 cells from p38 and JNK signaling pathway induced cell death, and further proved that RES plays an important role in protecting HLEB-3 cells against oxidative stress.

In conclusion, the present study suggested that RES could significantly reduce H_2_O_2_ induced intracellular ROS accumulation in human lens epithelial cells and protect the cells against H_2_O_2_ induced apoptosis. Furthermore we also showed that RES reduced H_2_O_2_ induced p38 and JNK activation, indirectly prevented HLEB-3 cells from p38 and JNK signaling pathway induced cell death. Our findings indicated that one of the underlying mechanisms might be linked to the increase of catalase, SOD-1, and HO-1 expression in HLEB-3 cells. Therefore RES, as a potent antioxidant agent and a natural compound found in several plants, may be exploited as a potentially useful method for cataract prevention. Further clarification of the molecular mechanism of RES involved in HLEB-3 cells may lead to the development of therapeutic strategies to prevent and or delay cataractogenesis in both humans and animals.
